# *TERT* promoter mutation is an objective clinical marker for disease progression in chondrosarcoma

**DOI:** 10.1038/s41379-021-00848-0

**Published:** 2021-06-09

**Authors:** Yifan Zhang, Yi Chen, Chen Yang, Nelly Seger, Asle C. Hesla, Panagiotis Tsagkozis, Olle Larsson, Yingbo Lin, Felix Haglund

**Affiliations:** 1 Department of Oncology-Pathology, Karolinska Institutet, Stockholm, Sweden; 2 Department of Clinical Pathology and Cytology, Karolinska University Hospital Solna, Stockholm, Sweden; 3 Department of Molecular Medicine and Surgery, Karolinska Institutet, Stockholm, Sweden; 4 Department of Orthopedic Surgery, Karolinska University Hospital Solna, Stockholm, Sweden

**Keywords:** Tumour biomarkers, Bone cancer

## Abstract

Chondrosarcomas are the second most common malignant bone tumor. Activating promoter mutations in *telomerase reverse transcriptase (TERT)* was recently described by us and others as a frequent mutation in high-grade chondrosarcoma. In this study, we investigate the prognostic significance of *TERT* promoter mutations in 241 chondrosarcomas from 190 patients collected over 24 years (1994–2017). The *TERT* promoter was sequenced after microdissection of 135 chondrosarcomas from 106 patients in addition to data from our previous cohort. The *TERT* promoter mutation at −124 C > T was found in 45% of all patients and was significantly associated (*p* > 0,001) with higher tumor grade, shorter metastasis-free survival, and disease-specific survival. Additionally, *TERT* promoter-mutated tumors were associated with a more aggressive metastatic pattern. Shorter survival was observed in patients with wild-type primary tumors who developed a mutated metastasis indicative of tumor progression. Primary tumor genetic heterogeneity and altering mutational status between nonsynchronous metastatic lesions suggests that chondrosarcoma is a multiclonal disease progressing through a branching evolution. Conclusion: *TERT* promoter mutation seems to be a central event in chondrosarcoma progression with association to metastatic disease and disease-related mortality. As an easily analyzed marker, there is future potential to utilize *TERT* promoter mutation status as a prognostic marker and investigate telomerase-targeted therapy in chondrosarcomas.

## Introduction

Chondrosarcoma (CS) is the 2nd most common sarcoma of bone origin, with a yearly incidence of three cases per million individuals. They are malignant cartilage-forming tumors, frequently localized in the femur, humerus, pelvis, and ribcage, but may arise in all bones. There are multiple subtypes of CS, 85% are conventional CS cases and 15% are rarer subtypes such as dedifferentiated, clear cell, and mesenchymal CS. Based on the histomorphology, they are classified into grades 1−3 (low-, intermediate-, and high grade) or as dedifferentiated CS if a dedifferentiated tumor component is present [1–3].

Most chondrosarcomas are primary tumors, but a subset is secondary and arising from preexisting lesions such as osteochondromas and enchondromas. A handful of mutations have been identified in both enchondromas and chondrosarcomas; one example is *IDH1* and *IDH2* genes, indicating that these mutations occur early in tumorigenesis. Some syndromes such as Ollier and Maffucci diseases have mosaic mutations in *IDH1* and *IDH2*, and thus a predisposition for multiple enchondromas [4, 5].

Human telomerase reverse transcriptase (*TERT*) gene promoter mutations have been observed in multiple types of malignant tumors [6]. It is believed that telomerase activity is induced by *TERT* gene overexpression, as transcription factor-binding motifs are generated as a result of the mutation [7, 8]. The maintenance of telomeres constitutes one of the hallmarks of cancer [9], and the presence of *TERT* mutation is generally associated with disseminated disease [10]. This mutation has previously been described in CS [11, 12], and studies on chondrosarcoma cell lines have indicated that telomerase inhibition may potentially sensitize drug-resistant CS cells to chemotherapy [13]. This is of importance as CS is highly chemo- and radiotherapy-resistant [14, 15], with surgical resection as the only curative treatment.

In a previous cohort, our group identified frequent *TERT* promoter-activating mutations in CS. Of 87 patients with CS, the mutation was found in 23 patients (26%) and identified as a strong negative prognostic factor with a significant correlation to disease progression in terms of metastasis and mortality. In addition, the mutation was also significantly associated with high tumor grade and was present in about half of our high-grade cases. In tumors with varying low- and high-grade areas, mutations were found in subclonal areas with aggressive morphology, while low-grade areas remained *TERT* promoter wild type [11].

Our previous cohort studies on *TERT* promoter mutation in CS are limited. In this study, we investigate *TERT* promoter mutations in CS and its prognostic implications in a large cohort of 190 patients.

## Materials and methods

### Patient and tumor samples

**Fig. 1:**
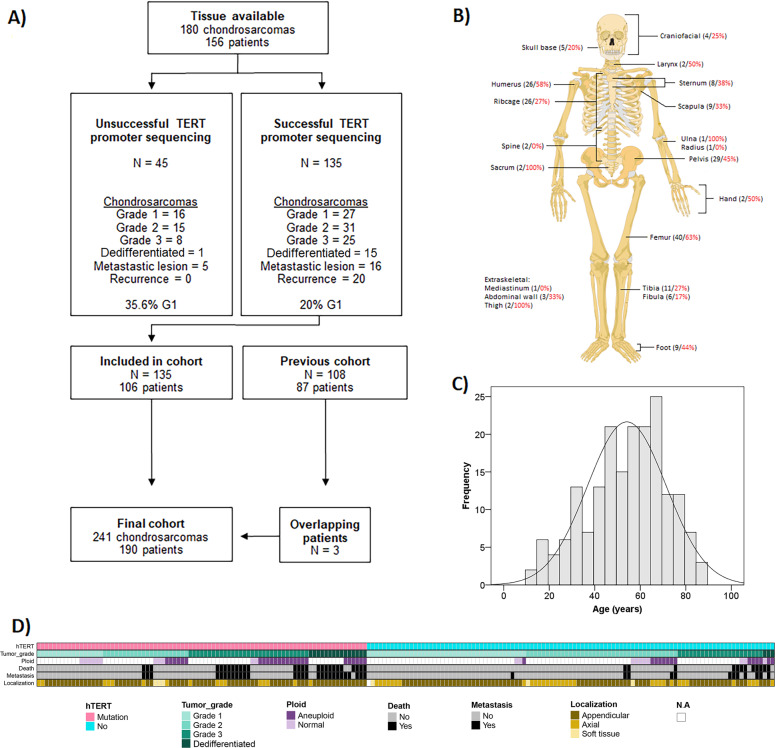
Study inclusion process, clinical and tumor characteristics. **A** Flow-chart showing the inclusion process. All cases with available tissue were included in the study. The *TERT* promoter was successfully sequenced in 75% of these cases. In unsuccessful cases, the proportion of grade 1 lesions was larger 35.6% versus 20%, in the successfully sequenced group, potentially linked to the paucicellular often seen in low-grade chondrosarcomas. **B** Anatomical localization of chondrosarcomas (total no. of tumors in localization (black)/% *TERT* promoter-mutated tumors (red)). **C** Age distribution of the 190 chondrosarcoma patients. **D** Schematic overview of clinical and tumor characteristics of the 190 patients.

Chondrosarcomas were identified by searching the archives of the Department of Clinical Pathology at the Karolinska University Hospital (Fig. 1A). Sequencing data from 87 patients in our previous study of *TERT* promoter mutations in a cohort of chondrosarcomas were integrated into the current study, bringing the total to 241 tumors from 190 patients with conventional CS. Clinical data and pathology evaluations were collected from the patients’ digital records. Tumor grade followed the WHO2020 classification [3] (based on the classification from 1985 as suggested by Mirra et al.) [16]. If more than one histological grade was described in a patient, the highest grade was used for the purpose of statistical comparison. We also collected data on tumor anatomical site, size, DNA ploidy analysis (when available), information of any preexisting lesion, or hereditary context.

### Ethical permission

The study was approved by the local ethical board (*Regionala Etikprövningsnämnden Stockholm*, registration number 2013 1979-31).

### DNA extraction and TERT promoter sequencing

In the previously analyzed cohort, full-block sections were sequenced from tumors in the earlier cohort. Since tumors were found to harbor subclonal *TERT* promoter mutations in histologically aggressive areas, we aimed to increase the sensitivity by making use of tumor microdissection. Multiple areas were identified and successfully sequenced in 25 (24%) of the cases. Routine hematoxylin-eosin-stained slides were reviewed, and from each tumor, an area representing the highest morphological grade was selected and manually microdissected from formalin-fixed paraffin-embedded (FFPE) blocks using 1-mm circular punch biopsies in clean conditions. Between 1 and 3 punch biopsies from each tumor were investigated. DNA was extracted using a QIAamp FFPE extraction kit (QIAgen). Sanger sequencing was carried out as previously described [11] with few exceptions. In short, polymerase chain reaction (PCR) with M13-tagged primers targeting the *TERT* promoter was done using a touch-down thermocycling protocol. Reactions were run in 50-µl volumes using AmpliTaqGold 360 Mastermix (Thermo Fisher Scientific, Waltham, MS, USA) and >10 ng of gDNA. PCR products were purified using ExoZap-IT PCR Product Cleanup Reagent (Thermo Fisher Scientific, Waltham, MS, USA) or spin columns (QIAquick PCR Purification Kit, Qiagen, Hilden, Germany). Purified PCR products were sequenced by GATC Biotech (Cologne, Germany) using M13 primers, and chromatograms were manually interpreted using 4Peaks Software (version 1.7.1., Mekentosj).

### Statistics

Time to event was defined as the timeframe between the date of surgery and the date of the event. Differences in overall- and metastasis-free survival (MFS) were calculated with the Kaplan−Meier method. Differences between groups were calculated with the log-rank test. A two-sided Fisher’s exact test and Chi-squared test were used to compare categorical variables. Mann−Whitney U test was used to compare categorical and continuous variables. A *p*-value of <0.05 was defined as statistically significant. To ascertain the association between clinical features and prognosis in CS, univariate and multivariate analysis was performed using the Cox regression model in R package “survival”.

## Results

### Clinical data and patient demographics

**Fig. 2:**
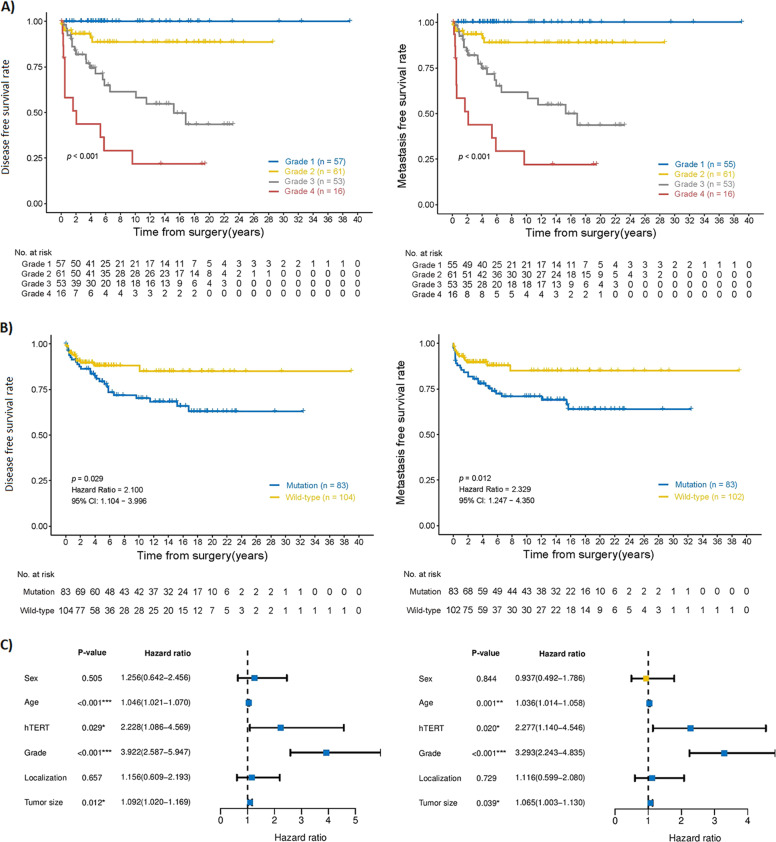
Disease-specific and metastasis-free survival in chondrosarcoma patients. Kaplan−Meier curve of disease-specific survival probability (left) and metastasis-free survival (MFS) (right) in the presence of **A** histological tumor grade and **B** *TERT* mutation status. **C** Univariate analysis of disease-specific survival (right) and MFS (right).

A total of 241 CS from 190 patients were identified, including the 87 patients from the previous cohort [11] (Fig. 1A and Table 1). At follow-up (average 8.5 years, min−max 0.1–32), 39 patients (21%) had developed distant metastasis (average time to metastasis after 2.8 years, min−max 0–15.7 years), and 38 (20%) patients had died of their disease (Table 1). The patient age had a normal distribution centered around the median age of 55 years (average 54 years, min−max 12–85 years) (Fig. 1C) with a 1,12:1 male-to-female ratio. A hereditary background with Ollier disease, Maffucci disease or multiple exostoses was present in seven patients, and an additional eight patients had a preexisting lesion of either fibrous dysplasia or exostosis. Tumor localization was more common in the appendicular skeleton compared with axial (Table 1). The anatomical site had no significant difference regarding metastasis-free and overall survival (Supplementary Fig. 1). As expected, higher tumor grade was by itself significantly associated with reduced metastasis-free and overall survival (Fig. 2A).

**Table 1:** Clinical characteristics and *TERT* promoter mutation status in chondrosarcoma patients.

	New cohort *TERT* promoter				Old + new cohort *TERT* promoter			
	All cases	Wild-type	Mutated	*P*-value	All cases	Wild-type	Mutated	*P*-value
Total number of patients	106	41	65		190	105	86	
Chondrosarcoma grade								
*Grade 1*	23	9	14	0.035*	58	41	17	<0.0001***
*Grade 2*	36	20	16		61	39	22	
*Grade 3*	29	9	20		53	22	31	
*Dedifferentiated*	18	3	15		18	3	15	
Tumor size (cm)								
*Average* *±* *SD (min−max)*	9.2 ± 4.7 (2.9–20)	8.7 ± 4.7 (3.3–19)	9.6 ± 4.8 (2.9–20)	*0.588*	8.6 ± 5.5 (1–36)	8.4 ± 4.6 (1–36)	9.0 ± 4.6 (2.9–20)	*0.190*
Patient age (years)								
*Average* *±* *SD (min−max)*	54 ± 16.8 (12–85)	57 ± 15.8 (24–85)	51 ± 17.1 (12–77)	*0.198*	54 ± 17.4 (12–88)	54 ± 18 (17–88)	54 ± 16.5 (12–85)	*0.749*
Sex								
*Male*	53	22	31	*0.839*	102	60	42	*0.309*
*Female*	53	20	33		88	44	44	
Skeletal location (primary tumor)								
*Axial*	28	17	11	0.012*	49	33	16	*0.119*
*Appendicular*	73	22	51		134	68	66	
*Extraskeletal*	4	1	3		6	3	3	
*N.A*	1	1	0		1	1	0	
Metastasis								
*Yes*	30	8	22	*.126*	39	12	27	0.001**
*No*	76	33	43		151	93	58	
Local recurrence								
*Yes*	30	14	16	*0.390*	50	29	21	*0.390*
*No*	76	27	49		140	77	63	
Death at follow-up								
*Dead*	40	14	26	*0.681*	51	18	33	0.001**
*Alive*	66	27	39		139	87	52	
Dead of disease								
*Chondrosarcoma*	30	9	21	*0.277*	38	12	26	0.001**
*Alive or other death*	76	32	44		152	94	58	
Follow-up time (years)								
*Average* *±* *SD (min−max)*	12 ± 8.2 (0.07–32)	11.4 ± 8.1 (0.33–29)	12.7 ± 8.2 (0.07–32)	*0.991*	8.5 ± 8.2 (0.04–32)	6.9 ± 7.0 (0.04–31)	10.7 ± 8.0 (0.07–32)	*0.002*
Hereditary background								
*Mb Ollier*	3	1	2		4	1	3	
*Mb Mafficci*	0	0	0		1	0	1	
*Multiple exostosis*	1	0	1		2	1	1	
Preexisting lesion								
*Fibrous dysplasia*	1	0	1		1	0	1	
*Exostosis*	1	0	1		7	5	2

### TERT promoter mutation is common in conventional chondrosarcomas

Sanger sequencing of the *TERT* promoter was successful in 135 tumors (Fig. 1A). In comparison, the remaining unsuccessful cases consisted of a higher degree of low-grade tumors, likely explained by low tumor cellularity leading to low DNA yields.

The previously characterized—124 C > T *TERT* promoter mutation was detected in 45% of the patients, and no other *TERT* promoter mutations were identified. There was a significant association between *TERT* promoter mutation status and higher chondrosarcoma grade (*p* < 0.0001), as well as metastasis (*p* < 0.001), and both overall (*p* < 0.001) and disease-specific mortality (*p* < 0.001), but not with local recurrence (*p* = 0.390) (Table 1). Kaplan−Meier curves showed a clear separation between *TERT* promoter wild-type and mutated cases (Fig. 2B). There was no obvious association or significant difference in *TERT* promoter mutation frequency at different anatomical sites, nor any significant difference between tumors of the appendicular and axial skeleton (*p* = 0.119) (Table 1).

Univariable regression analysis identified tumor grade, *TERT* promoter mutations, patient age, and tumor size as independent negative prognostic variables (Fig. 2C). After multivariable regression including these four variables, only tumor grade remained significant (Supplementary Fig. 2), indicating the strong association between *TERT* promoter mutation and tumor grade. The *TERT* promoter status did not seem to segregate the risk of metastasis within any given histological grade, which was also supported by Kaplan−Meier curves stratifying for tumor grade (data not shown).

We also performed subgroup analyses using univariable regression (Supplementary Fig. 3) and identified that *TERT* promoter mutations had a higher hazard ratio for both metastasis and death in older patients (>55 years/over the median age) (Supplementary Fig. 4). Further analysis revealed that the younger group held no dedifferentiated tumors and much fewer metastasis or death events (11/94) compared with the older group (28/94 metastasis and 26/94 patients dead-by-chondrosarcoma), indicating a risk for a type-II error. Thus, an even larger cohort would be required to determine the potential difference in HR between these two age groups.

### TERT promoter mutation and differences in metastatic disease

**Fig. 3:**
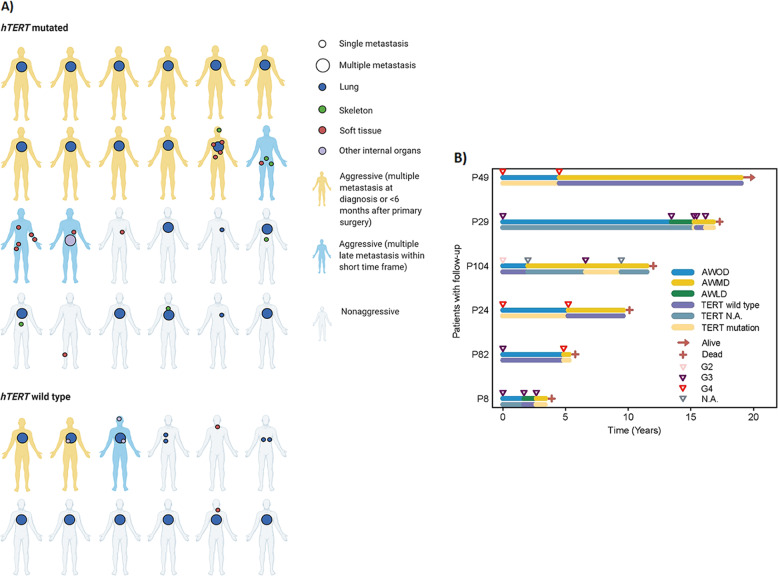
Course of disease and longitudinal data in presence of *TERT* mutation status. **A** Illustration of metastasis locales and course of disease in 38 patients with metastatic disease in the presence of *TERT* mutation status. **B** Swimming plot illustrating longitudinal data of patients with metastatic disease and altering *TERT* promoter status. For each patient, the upper line indicates disease status (AWOD alive without disease, AWMD alive with metastatic disease, AWLD alive with local disease) and the arrows indicate tumor grade.

A total of 39 patients developed metastasis, of which 24 harbored *TERT* promoter mutation; 36 patients had more detailed clinical data available (Fig. 3A). Metastasis at diagnosis or within six months of surgery was seen in 13 cases (11 were *TERT* promoter-mutated). A late but aggressive metastasis pattern (multiple metastases within a short time frame) suggestive of transformation was seen in four patients (three *TERT* promoter-mutated). Importantly, among patients that developed metastasis, there was no associated age difference (*p* = 0.927) or apparent diagnostic delay (as determined when reviewing the files) associated with *TERT* promotor status that could explain this difference. After reviewing the patients’ files, it thus became clear that *TERT* promoter-mutated tumor presented with a more aggressive metastatic pattern.

### Chondrosarcomas are capable of branching evolution

From the patients that developed metastasis, six patients had altering *TERT* promoter status between tumors. Longitudinal plotting of the patient and sequencing data showed a tendency for shorter survival in those patients who had a wild-type primary tumor that presented with a mutated metastasis, in comparison to those who developed wild-type metastasis (Fig. 3B). In addition, one patient had multiple metastases that were either *TERT* wild type or mutated (confirmed by a second independent round of DNA isolation and sequencing). These data suggest that mutated metastases may arise from wild-type primary tumors; CS are frequently found to harbor subclonal mutations, which together with the fact that both wild-type and mutated metastases arise in one individual patient suggest that CS may develop in a branching evolution.

### Multiple-sample sequencing confirms the subclonal nature of TERT promoter mutations in conventional chondrosarcomas

A subset of patients had multiple samples for sequencing (Table 2). The tumors that underwent multiple sample sequencing had a higher percentage of identified *TERT* promoter mutations. The method of histological evaluation and selecting punch biopsies of higher-grade areas were more successful in identifying mutations compared with sequencing whole-tissue sections (Table 2). This suggests that there is a significant tumor heterogeneity with *TERT* promoter-mutated subclonal populations, but that these areas can be morphologically identified as areas with higher tumor grade.

**Table 2:** Percentage of *TERT* promoter mutated tumors using multiple sample sequencing compared to overall results. In the previous cohort whole tissue sections from a single FFPE block were sequenced, but a few samples were sequenced using punch biopsies from high-grade areas; the new cohort used punch biopsies from the worst morphological area for all cases.

	No. of patients	*TERT* mutated (%)	*TERT* wild type (%)
New cohort			
*Multiple samples*	25	72	28
*Overall*	106	61	39
Old cohort			
*Multiple samples*	15	33	67
*Overall*	87	26	74
Overlapping patients			
*Multiple samples*	3	67	33
*Overall*	3	67	33
Total			
*Multiple samples*	43	57	43
*Overall*	190	45	55

Additionally, all dissected tumor areas were microphotographed and reviewed in both a blinded and unblinded fashion (without and with knowledge of the tumor *TERT* promoter status), but aside from overall tumor grade, there was no specific morphological pattern (including cellularity, chondrocyte shape, nuclear format, and matrix appearance) clearly associated with *TERT* promoter mutations (data not shown).

### Nomogram for prediction of MFS

**Fig. 4:**
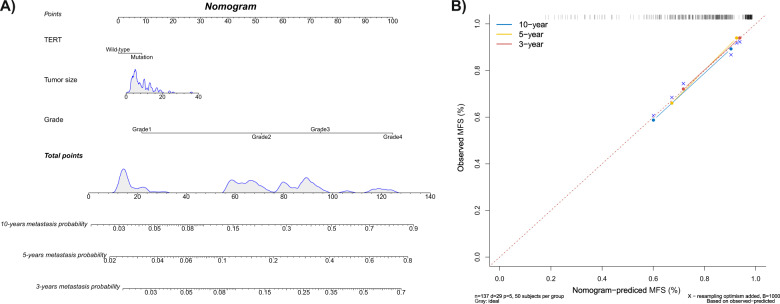
Nomogram for prediction of metastasis-free survival. **A** Nomogram for predicting metastasis probability in patients with chondrosarcoma. **B** Internal calibration plot for 3-, 5-, and 10-year metastasis-free survival (MFS).

*TERT* mutation status, tumor size, and tumor grade were independent prognostic predictors for MFS in CS patients (Fig. 4A). A calibration plot demonstrates the nomogram-predicted probability and actual MFS probability as well-fitting on internal and external validation (Fig. 4B).

## Discussion

In this study, we aimed to validate the prognostic value of *TERT* promoter sequencing by analyzing the vast majority of chondrosarcomas treated in our institution over a period of 27 years. While decalcification and paucicellularity of low-grade tumors posed a methodological challenge, 75% of all identified tumors were successfully sequenced. This resulted in a final cohort of 190 patients where the previously characterized—124 C > T *TERT* promoter mutation was identified in as many as 45% of the tumors. In line with our previous findings, *TERT* promoter mutations significantly associated with high tumor grade, metastasis, and disease-related mortality. By detailed review of patient records, we were able to identify that *TERT* promoter mutations were significantly associated with a more aggressive clinical course, including signs of transformation, early metastasis, or a late but aggressive metastasis pattern. Furthermore, shorter disease-specific survival was observed in those with wild-type primary tumors who later developed *TERT-*mutated metastasis compared with those with metastatic disease who remained wild type. Our data suggest that subclonal *TERT* promoter mutations may be a central event for tumor progression in chondrosarcoma, and its identification might be helpful to identify patients with higher risk of tumor metastasis. In clinical routine pathology, the identification of *TERT* promoter mutations may also help verify if the suspicious cell population is truly neoplastic, rather than tumor-associated reactive spindle cell areas frequently observed in chondrosarcomas.

The new cohort showed some differences in the distribution of *TERT* promoter mutations as compared with the previously published cohort. These differences could in part be explained by the different sequencing method, the wider inclusion criteria, or the much longer follow-up in the current study. Not surprisingly, a higher rate of *TERT* promoter mutations was identified using multiple-sample sequencing. In addition, in our longitudinal data, we observed mutated metastasis stemming from wild-type primary tumors. Tumor heterogeneity should be considered when analyzing for *TERT* promoter mutations; punch biopsies of the highest-grade areas chosen by histological evaluation seem to yield the best results in capturing *TERT* promoter-mutated areas, but other methods such as digital droplet PCR (ddPCR) or targeted NGS could potentially have higher sensitivity. In future studies, ddPCR would be of interest to investigate if a quantitative measure of mutated alleles would correlate with tumor grade and prognosis.

In conclusion, in the hitherto largest study of *TERT* promoter mutations in conventional CS, we observed that *TERT* promoter mutation was a negative prognostic marker, with a clear, significant correlation to high tumor grade, metastatic disease, and mortality. Our results indicate that CS is capable of branching evolution, which has not been previously reported. *TERT* promoter mutations are easily analyzed and hold promising potential as an objective clinical prognostic marker, as well as a potential therapeutic target including telomerase-targeting approaches. In addition, *TERT* promoter mutation analysis could serve as a complement to routine pathology to distinguish high-grade areas in cases where the morphology is inconclusive.

## Supplementary information

Supplementary figures

## Data Availability

The datasets used and/or analyzed during the current study are available from the corresponding author on reasonable request.
