# Supplementary material for: TERT promoter mutation is an objective clinical marker for disease progression in chondrosarcoma
Source: Mod Pathol. 2021 Jun 9;34(11):2020–7. doi: 10.1038/s41379-021-00848-0 (PMC8514332; doi:10.1038/s41379-021-00848-0)
Supplement: Supplementary file 1 — Supplementary figures [file 41379_2021_848_MOESM1_ESM.docx]

**Supplementary figures**

**
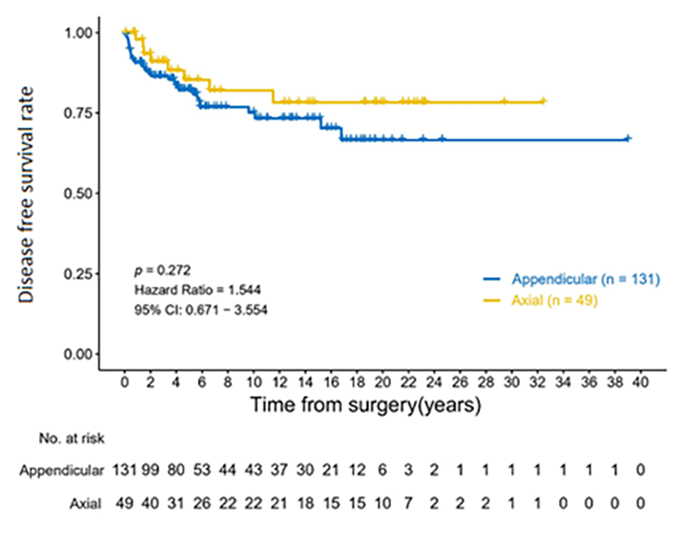
**

**
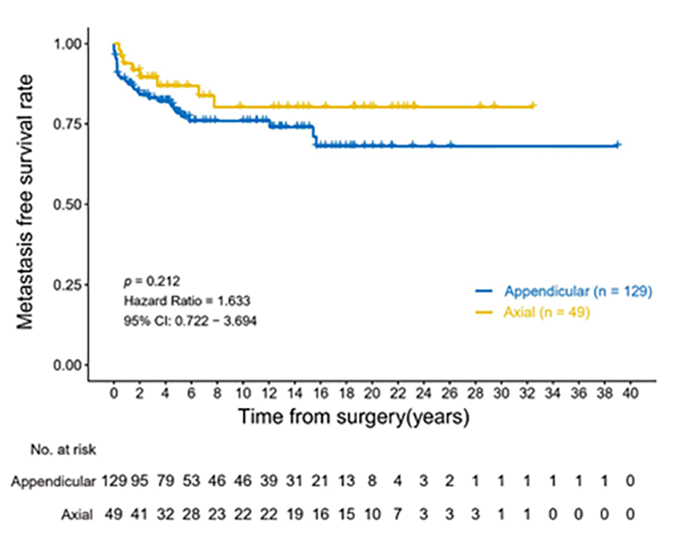
**

**Supplementary figure 1**: Kaplan-Meier curve for disease-free survival (top) and metastasis-free survival (bottom) with regard to tumor site.

**
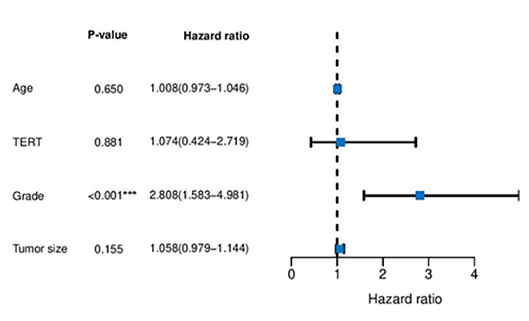
**

**
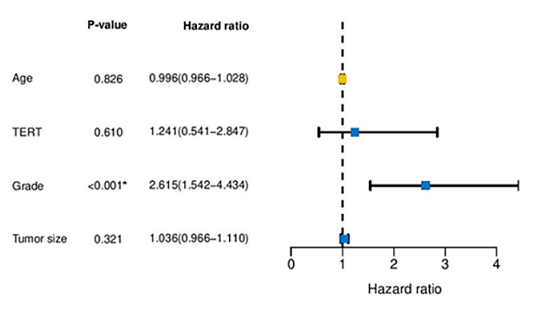
**

**Supplementary figure 2**: Multivariable regression analysis of disease-specific survival (top) and metastasis-free survival (MFS) (bottom).

**
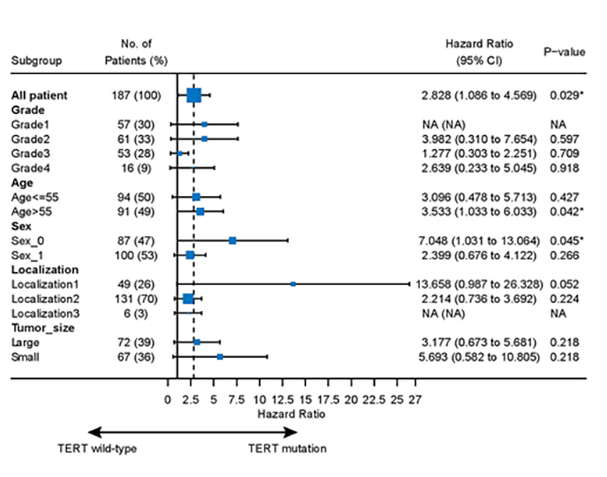
**

**
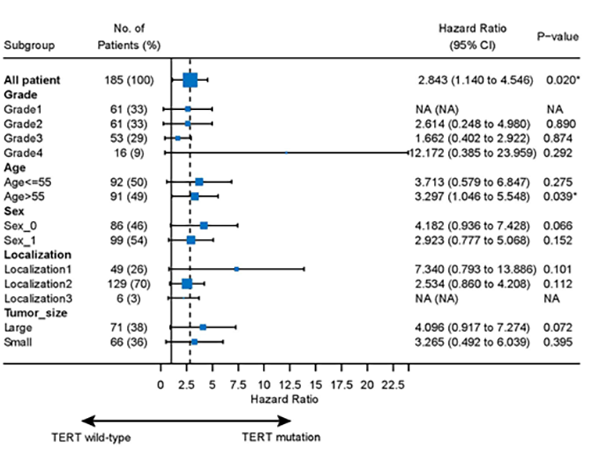
**

**Supplementary figure 3**: Subgroup analysis for of disease-specific survival (top) and and metastasis-free survival (bottom).


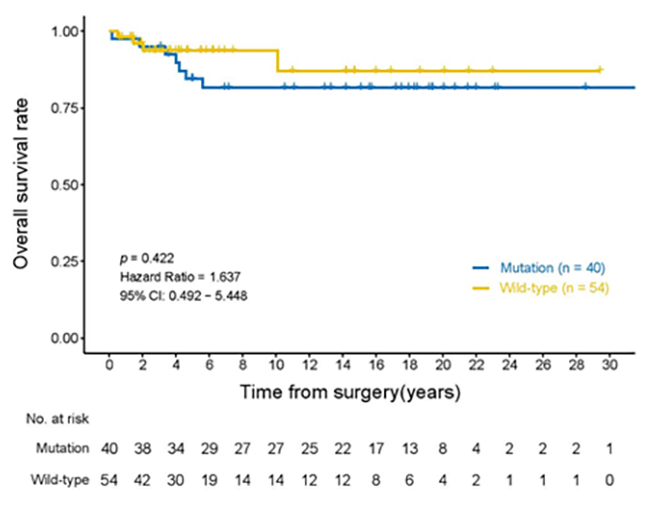


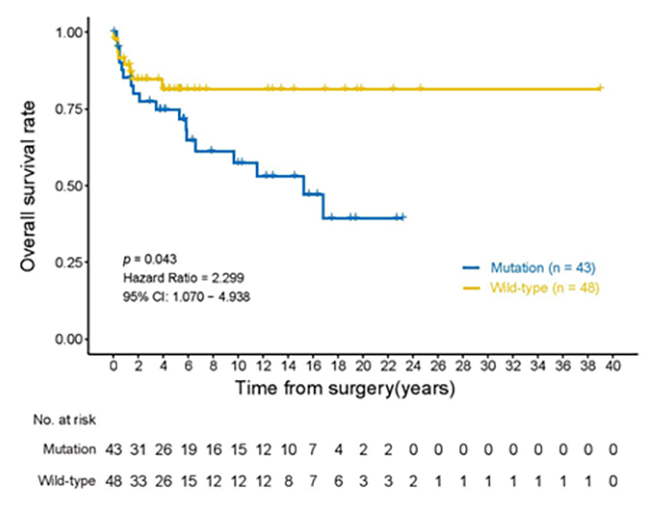


**Supplementary figure 4**: Kaplan-Meier curve of overall survival (OS) for patients with age ≤ 55 (top) and > 55 (bottom).
